# Supplementary material for: The proton channel OTOP1 is a sensor for the taste of ammonium chloride
Source: Nat Commun. 2023 Oct 5;14:6194. doi: 10.1038/s41467-023-41637-4 (PMC10556057; doi:10.1038/s41467-023-41637-4)
Supplement: Supplementary file 1 — Supplementary Information [file 41467_2023_41637_MOESM1_ESM.pdf]

The Proton Channel OTOP1 is a Sensor for the Taste of Ammonium Chloride

Ziyu Liang[1,2,*], Courtney E. Wilson[3,*], Bochuan Teng[1,2,4], Sue C. Kinnamon[3] and Emily R. Liman[1,#]

1. Section of Neurobiology, Department of Biological Sciences, University of Southern California, Los Angeles, CA, 90089, USA.
2. Program in Neuroscience, University of Southern California, Los Angeles, CA, 90089, USA.
3. Department of Otolaryngology, University of Colorado Medical School, 12700 E 19(th) Avenue, MS 8606, Aurora, CO 80045, USA.
4. Present address: Division of Biology and Biological Engineering, California Institute of Technology, Pasadena, CA, USA.

*These authors contributed equally

[#] Correspondence: liman@usc.edu

Supplementary Material

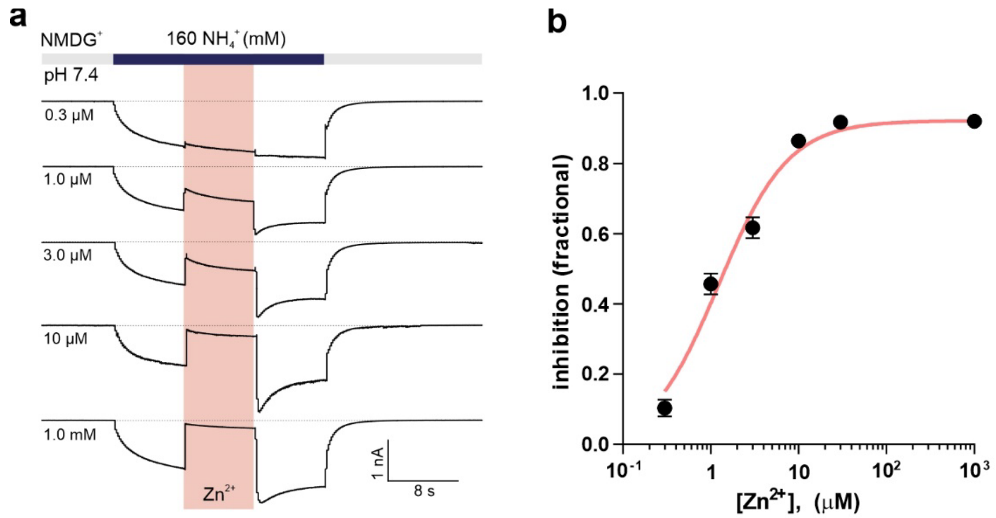

Supplementary Figure.1 Dose-dependent inhibition of NH_4Cl-induced current in mouse OTOP1 by Zn^{2+}.

(a) Representative trace of currents in mouse OTOP1 expressing HEK-293 cells elicited in response extracellular 160 mM NH_4Cl were inhibited in a dose-dependent manner by Zn^{2+} (pink bar, concentrations indicated as in the figure). **(b)** Average data from experiments as in (A) (mean ± SEM). The data were fit with a Hill equation with an IC_{50} of 1.3 µM and a Hill coefficient of 1.1. n = 2 cells for 10 µM, n = 3 cells for 30 µM, n = 7 cells for 0.3 and 3.0 µM, n = 8 for 1.0 µM and 1 mM.

Supplementary Table 1

Oligonucleotides		
Whole exon 1 genotyping forward: GGAGCCAGGAGCAACGTGC	IDT	N/A
Whole exon 1 genotyping reverse: CTGCTCAGAGTCTCCGCCAGC	IDT	N/A
WT-specific allele genotyping forward: TGGAGGAGAGCAGGATCTGAGG	IDT	N/A
WT-specific allele genotyping reverse: AGTCTCCGCCAGCTTCTGCG	IDT	N/A
Mutant (-38)-specific allele genotyping forward: TGGAGGAGAGCAGGATCTGAGG	IDT	N/A
Mutant (-38)-specific allele genotyping reverse: GTCTCCGCCAGCTTCTGGA	IDT	N/A
mOTOP1 K187A mutation forward: CTGGGGCCATGCTGCTGATATCATCATG	IDT	N/A
mOTOP1 K187A mutation reverse: GATGATATCAGCAGCATGGCCCCAGAGG	IDT	N/A
mOTOP1 R292A mutation forward: GAACATCGGGGCCAGAGTGGAC	IDT	N/A
mOTOP1 R292A mutation reverse: CCACTCTGGCCCCGATGTTC	IDT	N/A
mOTOP1 K527A mutation forward: CAGGGCGGCATGGCCAGGAGGCTTC	IDT	N/A
mOTOP1 K527A mutation reverse: CCTCCTGGCCATGCCGCCCTG	IDT	N/A
mOTOP1 R528A mutation forward: GCATGAAGGCCAGGCTTCTCAG	IDT	N/A
mOTOP1 R528A mutation reverse: GAAGCCTGGCCTTCATGCCGCCCTG	IDT	N/A
