# Supplementary material for: The proton channel OTOP1 is a sensor for the taste of ammonium chloride
Source: Nat Commun. 2023 Oct 5;14:6194. doi: 10.1038/s41467-023-41637-4 (PMC10556057; doi:10.1038/s41467-023-41637-4)
Supplement: Supplementary file 3 — Reporting Summary [file 41467_2023_41637_MOESM3_ESM.pdf]

nature portfolio

Corresponding author(s): Emily R. Liman

Last updated by author(s): 09-4-2023

Reporting Summary

Nature Portfolio wishes to improve the reproducibility of the work that we publish. This form provides structure for consistency and transparency in reporting. For further information on Nature Portfolio policies, see our Editorial Policies and the Editorial Policy Checklist.

Statistics

For all statistical analyses, confirm that the following items are present in the figure legend, table legend, main text, or Methods section.

n/a	Confirmed	
☐	☒	The exact sample size (*n*) for each experimental group/condition, given as a discrete number and unit of measurement
☐	☒	A statement on whether measurements were taken from distinct samples or whether the same sample was measured repeatedly
☐	☒	The statistical test(s) used AND whether they are one- or two-sided<br>*Only common tests should be described solely by name; describe more complex techniques in the Methods section.*
☐	☒	A description of all covariates tested
☐	☒	A description of any assumptions or corrections, such as tests of normality and adjustment for multiple comparisons
☐	☒	A full description of the statistical parameters including central tendency (e.g. means) or other basic estimates (e.g. regression coefficient) AND variation (e.g. standard deviation) or associated estimates of uncertainty (e.g. confidence intervals)
☐	☒	For null hypothesis testing, the test statistic (e.g. *F*, *t*, *r*) with confidence intervals, effect sizes, degrees of freedom and *P* value noted<br>*Give P values as exact values whenever suitable.*
☒	☐	For Bayesian analysis, information on the choice of priors and Markov chain Monte Carlo settings
☒	☐	For hierarchical and complex designs, identification of the appropriate level for tests and full reporting of outcomes
☒	☐	Estimates of effect sizes (e.g. Cohen's *d*, Pearson's *r*), indicating how they were calculated

Our web collection on statistics for biologists contains articles on many of the points above.

Software and code

Policy information about availability of computer code

Data collection	PClamp 8.2 (Molecular Devices), SimplePCI (HCImage)
Data analysis	Prism (Graphpad) vers 9.5.1, Clampfit 10.7 (Molecular Devices), Origin 6.1 (Origin Lab Corp), Pymol 2.5.2 (Schrodinger Inc)

For manuscripts utilizing custom algorithms or software that are central to the research but not yet described in published literature, software must be made available to editors and reviewers. We strongly encourage code deposition in a community repository (e.g. GitHub). See the Nature Portfolio guidelines for submitting code & software for further information.

Data

Policy information about availability of data

All manuscripts must include a data availability statement. This statement should provide the following information, where applicable:

- Accession codes, unique identifiers, or web links for publicly available datasets
- A description of any restrictions on data availability
- For clinical datasets or third party data, please ensure that the statement adheres to our policy

All data supporting the findings of this study are available within the paper and its Supplementary Information. The source data underlying Figure 1b-c, 1e, 2b, 2d, 2f, 3b-c, 3e, 3f, 4b, 5b-c, 6c, and Supplementary Figure 1b are provided as a Source Data file. The PDB structure of mOTOP1 can be found at https://alphafold.ebi.ac.uk/entry/Q80VM9

Research involving human participants, their data, or biological material

Policy information about studies with human participants or human data. See also policy information about sex, gender (identity/presentation), and sexual orientation and race, ethnicity and racism.

Reporting on sex and gender	N/A
Reporting on race, ethnicity, or other socially relevant groupings	N/A
Population characteristics	N/A
Recruitment	N/A
Ethics oversight	N/A

Note that full information on the approval of the study protocol must also be provided in the manuscript.

Field-specific reporting

Please select the one below that is the best fit for your research. If you are not sure, read the appropriate sections before making your selection.

[x] Life sciences   [ ] Behavioural & social sciences   [ ] Ecological, evolutionary & environmental sciences

For a reference copy of the document with all sections, see nature.com/documents/nr-reporting-summary-flat.pdf

Life sciences study design

All studies must disclose on these points even when the disclosure is negative.

Sample size	Sample size was determined to be consistent with the standard for the field. For electrophysiological recordings from single cells (transfected HEK cells or taste receptor cells), we attempted to obtain >3 independent separate cells (Tu et al., 2018). For gustatory nerve recording, we measured responses from 7-15 independent replicates (animals) per genotype/stimulus (Teng et al., 2019); For behavioral analyses, we measured responses from 8-37 independent replicates (animals) per genotype (Teng et al., 2019). A large number of WT mice were used as these were tested on the same days as animals from the three other genotypes.
Data exclusions	For behavior, animals and data exclusion were performed by an investigator blinded to genotype. Animals were tested with the same solution set for three consecutive days. Experimental days were excluded if the average licks to the control stimulus (artificial saliva), used to normalize data, was less than or equal to 20, which represents half the maximal licks an animal can perform in the alloted time. Only animals that completed two of the three days were included in the analysis.
Replication	For electrophysiological recordings from isolated cells, reproducibility was assessed by comparing responses measured over several days. All replications from independent cells are included in the data and the n is indicated in each figure legend. All data was found to be reproducible. A similar process was used to assess reproducibility of behavioral data and gustatory nerve recording. All replications from independent animals are included in the data and the n is indicted in each figure legend. All attempts at replication were successful. Note that outliers were not removed, except if they met data exclusion criteria described above.
Randomization	Randomization is not relevant to our study as animals or constructs were members of a group based on their genotype/sequence.
Blinding	For behavioral and nerve recording analyses, investigators were aware of the genotype of the mice. Measurements were made with automated systems, normalized to baseline for each animal, and thus could not be subject to experimental bias. Posthoc data exclusion was done by a separate investigator, blinded to genotype. For patch clamp recording, the investigator was not blinded to genotype. This was not relevant because these experiments included internal controls and all data was automatically recorded in such a manner that possible investigator bias could not affect the outcome.

Reporting for specific materials, systems and methods

We require information from authors about some types of materials, experimental systems and methods used in many studies. Here, indicate whether each material, system or method listed is relevant to your study. If you are not sure if a list item applies to your research, read the appropriate section before selecting a response.

Materials & experimental systems

n/a	Involved in the study
x	Antibodies
	x Eukaryotic cell lines
x	Palaeontology and archaeology
	x Animals and other organisms
x	Clinical data
x	Dual use research of concern
x	Plants

Methods

n/a	Involved in the study
x	ChIP-seq
x	Flow cytometry
x	MRI-based neuroimaging

Eukaryotic cell lines

Policy information about cell lines and Sex and Gender in Research

Cell line source(s)	HEK-293 (ATCC CRL-153) and PAC KO HEK-293 cells (gift of Zhaozhu Qiu)
Authentication	none of the cell lines were authenticated. They were provided directly by the manufacturer or by Dr. Qiu; For PAC KO cells, we confirmed that there were no outwardly rectifying currents indicative of PAC channels
Mycoplasma contamination	All experimental results were compared with untransfected cells, and health of the cells was monitored by visual inspection and viability in patch clamp recording. New cells were thawed as needed to maintain viability of the cell lines. These measures obviated the need to test for mycoplasma.
Commonly misidentified lines (See ICLAC register)	no commonly misidentified cell lines were used in this study

Animals and other research organisms

Policy information about studies involving animals; ARRIVE guidelines recommended for reporting animal research, and Sex and Gender in Research

Laboratory animals	All animal procedures were approved by the Institutional Animal Care and Use Committees of either the University of Southern California or the University of Colorado School of Medicine. The mouse strain PKD2L1-YFP is a BAC transgenic line in which the promoter of Pkd2l1 drives the expression of YFP as previously described29. The Otop1-KO mouse is a deletion of 38 bp from the 5' end of the Otop1 gene and was previously described31. Skn-1a-/- mice were generously provided by Ichiro Matsumoto (Monel Chemical Senses Center)46. Mice used in all experiments comprised both males and females. For nerve recording and behavior, mice were from the following genotypes: Otop1-/-, Skn-1a-/-, Otop1-/- x Skn-1a-/-, wildtype littermates and compatible background wildtype mice, ages between 6-30 weeks. Mice were housed at the University of Colorado Anschutz Medical Campus on a 12 hr light/dark cycle and had continual access to standard chow. Temperature was maintained at 72oC and >30% humidity. For isolated taste receptor cells, mice were from the following genotype: Otop1-/- and Otop+/+ in a PKD2L1-YFP background for cell identification. Mice were housed at the University of Southern of California on a 12 hr light/dark cycle and had continual access to standard chow. Temperature was maintained at 68oC – 74oC and humidity was 30-70%.
Wild animals	no wild animals were used in this study
Reporting on sex	Animals of both sexes were used in behavioral analyses, gustatory nerve recording and in experiments from isolated cells. There is no evidence in the literature for sex-differences in sour or ammonium taste and given the lack of statistical power did not do an analysis of sex as a biological factor.
Field-collected samples	No field collected samples were used in this study
Ethics oversight	All studies with animals were approved by the IACUC committees of the respective institutions (USC and University of Colorado, Denver).

Note that full information on the approval of the study protocol must also be provided in the manuscript.
